# The Association of Depressive Symptoms With Brain Volume Is Stronger Among Diabetic Elderly Carriers of the Haptoglobin 1-1 Genotype Compared to Non-carriers

**DOI:** 10.3389/fendo.2019.00068

**Published:** 2019-02-12

**Authors:** Abigail Livny, Michal Schnaider Beeri, Anthony Heymann, James Schmeidler, Erin Moshier, Ruth Tzukran, Galia Tsarfaty, Derek Leroith, Rachel Preiss, Laili Soleimani, Elizabeth Guerrero-Berroa, Jeremy M. Silverman, Barbara Bendlin, Andrew Levy, Ramit Ravona-Springer

**Affiliations:** ^1^The Joseph Sagol Neuroscience Center, Sheba Medical Center, Ramat Gan, Israel; ^2^Department of Diagnostic Imaging, Sheba Medical Center affiliated to Tel Aviv University, Tel Aviv, Israel; ^3^The Department of Psychiatry, Icahn School of Medicine at Mount Sinai, New York, NY, United States; ^4^Interdisciplinary Center, Baruch Ivcher School of Psychology, Herzliya, Israel; ^5^Department of Family Medicine, Tel Aviv University, Tel Aviv, Israel; ^6^Maccabi Health Services, Tel Aviv, Israel; ^7^Sackler Faculty of Medicine, Tel Aviv University, Tel Aviv, Israel; ^8^Department of Medicine, Icahn School of Medicine at Mount Sinai, New York, NY, United States; ^9^Geriatric Research Education and Clinical Center, William S. Middleton Memorial Veterans Hospital, Madison, WI, United States; ^10^Rambam Medical Center, Technion Institute of Technology, Haifa, Israel; ^11^Psychiatric Division, Sheba Medical Center, Ramat Gan, Israel

**Keywords:** haptoglobin genotype, type 2 diabetes, depression, brain volume, frontal lobe, white matter hyperintensities

## Abstract

**Aim:** Depression is highly prevalent in type 2 diabetes and is associated with lower adherence to medical treatments, worse glycemic control, and increased risk for diabetes-related complications. The mechanisms underlying depression in type 2 diabetes are unclear. The haptoglobin (Hp) genotype is associated with type 2 diabetes related complications including increased risk for cerebrovascular pathology and worse cognitive performance. Its relationship with depression is unknown. We investigated the role of Hp genotype on the association of depression with brain and white matter hyperintensities (WMH) volumes.

**Methods:** Depressive symptoms (measured with the 15-item Geriatric Depression Scale), brain MRI, and Hp genotypes, were examined in elderly subjects with type 2 diabetes [29 (13.8%) Hp 1–1 carriers and 181 (86.2%) non-carriers]. The interaction of Hp genotype with number of depressive symptoms on regional brain measures was assessed using regression analyses.

**Results:** The significant interactions were such that in Hp 1–1 carriers but not in non-carriers, number of depressive symptoms was associated with overall frontal cortex (*p* = 0.01) and WMH (*p* = 0.04) volumes but not with middle temporal gyrus volume (*p* = 0.43).

**Conclusions:** These results suggest that subjects with type 2 diabetes carrying the Hp 1–1 genotype may have higher susceptibility to depression in the context of white matter damage and frontal lobe atrophy. The mechanisms underlying depression in diabetes may differ by Hp genotype.

## Introduction

Depressive syndromes and symptoms, especially at sub-syndromal levels, are very common in old age (>65), reaching prevalence rates of 8–14% for minor depression (1), and 27% for depressive symptoms (2). Even in the absence of major depression, depressive symptoms in the elderly are associated with compromised aging, as reflected in poorer functional capacity and quality of life (3), and with increased morbidity and mortality (4). Depressive symptoms carry a poor prognosis, with lower response rates to antidepressant medications and tendency for recurrence, chronicity (4), and exacerbation (4). The risk for depression is doubled in the presence of type 2 diabetes [6], with a prevalence of 22–33% in elderly with diabetes (5), and is associated with lower adherence to medical treatments, worse glycemic control, and increased risk for diabetes-related complications (6). Development of better treatment and prevention strategies for depression in old age in the presence of diabetes requires better understanding of the underlying mechanisms.

Several mechanisms have been suggested for the association of depression and diabetes, including structural brain changes such as cerebral atrophy and cerebrovascular disease, which have been widely demonstrated in diabetes (7, 8).

Hp is an acute-phase protein, which binds to free hemoglobin and acts as an antioxidant (9). Two classes of alleles exist in humans: Hp 1 and Hp 2, yielding three genotypes (Hp 1–1, 2–1, and 2–2). Difference between these allelic protein products in structure and function are clinically relevant primarily to patients with diabetes, and less so to non-diabetic subjects (9). The Hp 2 allele is associated with increased risk for peripheral cardiovascular complication in diabetes (9), while the Hp 1 allele has been associated with increased risk for cerebrovascular morbidity (10), worse cognitive performance (11) and higher susceptibility of the hippocampus to the insults of poor glycemic control (12).

Previous studies have demonstrated brain volume abnormalities in subjects with depression and diabetes, specifically in the prefrontal cortex (13). Additionally, cerebrovascular abnormalities have been implicated as causal factors for depression in diabetes (14). There is scarce knowledge on role of these brain regions/abnormalities in depressive symptoms, which comprise the most prevalent clinical presentation of depression in the elderly (2)—rather than major depression—and specifically in diabetes. We have recently shown differential associations of the Hp genotype with hippocampal volume in diabetic elderly (12), but its contribution to the associations of depressive symptoms with brain volume is yet to be elucidated. Such differentiation may be of critical importance, as the efficacy of some interventions aimed at prevention of other diabetes-related complications (e.g., Vitamin E) differs by Hp genotype (15).

The present study aimed to assess whether the relationship of depressive symptoms with structural brain damage differs by Hp genotype in a cohort of elderly subjects with type 2 diabetes participating in the Israel Diabetes and Cognitive Decline (IDCD) study. Our study focused on the frontal lobe and on white matter hyperintensities (WMH) based on previous evidence consistently indicating structural and functional abnormalities of the frontal lobe region in depression (16), and on the role of vascular abnormalities in the form of WMH, specifically in old age depression (8).

## Methods

The IDCD is a collaboration of the Icahn School of Medicine at Mount Sinai, NY, the Sheba Medical Center, Israel, and the Maccabi Health Services (MHS), Israel. The study was approved by all three IRB committees.

### Subjects

Participants are elderly (≥65 years old) with type 2 diabetes, who are engaged in the IDCD study, a longitudinal investigation assessing the relationship of long-term type 2 diabetes characteristics and cognitive decline. The study was initiated in 2009. The present results are based on baseline data only; longitudinal follow ups are ongoing. The IDCD methods have been described in detail elsewhere (17). Briefly, IDCD participants were selected randomly from the ~11,000 elderly with diabetes listed in the diabetes registry of the MHS, the second largest HMO in Israel. The MHS diabetes registry was established in 1998 to facilitate disease management and to improve treatment, and has collected detailed information on laboratory, medication, and diagnoses. Entry criteria to the registry are any of the following: (1) HbA1c > 55.7 mmol/mol (7.25%), (2) Glucose >200 mg/dl on two exams more than three months apart, (3) purchase of anti-diabetic medication twice within 3 months supported by a HbA1c > 47.4 mmol/mol (6.5%) or Glucose > 125 mg/dl within half a year, (4) diagnosis of Type 2 diabetes (ICD9 code) by a general practitioner, internist, endocrinologist, ophthalmologist, or diabetes advisor, supported by a HbA1c > 47.4 mmol/mol (6.5%), or Glucose > 125 mg/dl within half a year.

Subjects are eligible for the IDCD study if they are: listed in the MHS diabetes registry, live in the central area of Israel, diagnosed as suffering from type 2 diabetes, are ≥65 years of age, identified as cognitively normal at baseline (based on a multidisciplinary weekly consensus conference), do not suffer from major medical, psychiatric, or neurological conditions that affect cognitive performance, have ≥3 HbA1c measurements in the diabetes registry, speak Hebrew fluently and have an informant.

Subject recruitment process has been described in detail previously (17). In short, the MHS team performs a thorough screening of the Diabetes Registry in order to identify potential subjects, excluding anyone with an ICD code for dementia/dementia subtypes, treatment with prescribed cholinesterase inhibitors, or with a major psychiatric or neurological condition (e.g., schizophrenia or Parkinson's disease) that could affect cognitive performance. After random selection of subjects, letters are sent by MHS to the primary care physicians, asking for permission to contact each patient regarding the study. If the doctors agree, letters are sent to the subjects briefly describing the study and saying that they will be contacted by phone in the following 2 week period. Then the MHS team calls the subjects and asks for their participation after determining that they are fluent in Hebrew and have a family member or caregiver who is willing to be an informant for the study. Subjects who are willing to participate in the study are assessed at their residence (or come to the Sheba Medical Center memory clinic, according to their preference) in two phases. First they are visited by a study physician who, after each subject has signed the informed consent form, performs medical, neurological, and geriatric assessments and draws blood for inflammatory markers (Il-6, CRP), Haptoglobin and APOE genotypes. In the second phase (optimally 2 weeks after the physician's visit), the subjects are visited by a neuropsychologist who administers a cognitive battery (described below), and questionnaires to the subject and informant for cognitive and functional impairment and for depression and behavioral disturbances characteristic of dementia. All subjects' cognitive data are discussed by a multidisciplinary consensus conference team in order to define the subjects' cognitive status (as cognitively normal, MCI, or dementia and their subtypes). If the subject is cognitively normal at baseline, the IDCD study has follow up interviews at 18 months intervals. Subjects, who are diagnosed as MCI at baseline, are not included in the study, however, subjects who convert from cognitive normal status to MCI during follow up, continue their participation in the study until conversion to dementia.

### Depressive Symptoms Assessment

The assessment of depression was performed using the 15- item version of the Geriatric Depression Scale (GDS) (18), a self-report scale designed to be simple to administer and not to require the skills of a trained interviewer. This questionnaire is suitable for large scale studies of depression and advantageous in the context of elderly with diabetes since it has little focus on somatic symptoms which could be confounded by diabetes symptoms such as neuropathy (18).

### Cognitive Assessment

A thorough neuropsychological battery that characterizes the breadth of cognitive functions is administered by experienced and certified interviewers which are blind to the diabetes related data. This battery covers four domains of cognitive functions: (1) Memory [Alzheimer's Disease Assessment Scale [ADAS] Word List Immediate Recall, Delayed Recall, and Recognition; (19)], (2) Attention/working memory [Diamond Cancellation, Digit Span forward, and backward (20)], (3) Executive functions [Trails Making Test A and B (21), Digit Symbol Substitution Test (20)], and (4) Semantic categorization [Similarities (20), Category Fluency (22)]. Composite measures of the four cognitive domains are calculated at baseline by converting each test score to a *z*-score and then summing the *z*-scores. An overall measure summing the scores was computed (overall cognitive score).

### MRI Procedures

All IDCD participants were offered to undergo a brain magnetic resonance imaging (MRI) scan. Those who agreed and did not have contraindications (e.g., claustrophobia, carriers of metallic grafts or pacemakers) were invited, by order of their expression of consent, to the Sheba Medical Center, diagnostic imaging department. Scans were performed using a 3 Tesla scanner (GE, Signa HDxt, v16VO2). High-resolution (1 mm^3^) images were acquired using a 3D inversion recovery prepared spoiled gradient-echo (FSPGR) T1-weighted sequence (TR/TE = 7.3/2.7s, 20o flip angle, TI 450 ms). The T1 weighted anatomical images for each subject were processed using the Voxel Based Morphometry [VBM (15)] toolbox, developed by Gaser (http://www.fil.ion.ucl.ac.uk/spm/ext/#VBMtools) and implemented in Statistical Parametric Mapping (SPM8) software. This procedure included automated iterative skull stripping, segmentation of the images into gray matter (GM), white matter (WM), and cerebrospinal fluid probability images, and spatial normalization of the GM images to a customized GM template in standard MNI (Montreal Neurological Institute) space. In order to optimize signal to noise, the GM maps were smoothed using an 8 mm Gaussian kernel. GM probability maps were thresholded at 0.1 to minimize inclusion of incorrect tissue types. Total intracranial volume (TICV) was calculated using the segmented and thresholded images (TICV = GM+WM+CSF). Based on our a-priori hypothesis, regions of interest (ROI) approach was used centered on the frontal cortex, specifically, the superior, middle, and inferior frontal gyri and the middle temporal gyrus identified by using the “Human Automated Anatomical Labeling (AAL) atlas” within the Wake Forest University PickAtlas (http://www.rad.wfubmc.edu/fmri) and extracted using the MarsBaR ROI toolbox as implemented in SPM8.

#### WMH Segmentation

We used SPM8 software (www.fil.ion.ucl.ac.uk/spm) and its VBM8 Lesion Segmentation Toolbox (LST), following previously described methods (23). The LST automated method for quantifying white matter damage is reliable and has been shown to have a high degree of agreement with manual delineation of WMH in fluid-attenuated inversion recovery images (23). The default LST settings were used with the exception of κ (k), a value indicating the threshold for the initial lesion mask. Visual inspection of the probability maps across participants by using various k values, to maximize sensitivity while reducing false positive results, indicated that a k = 0.15 was the optimal value for our sample images. This procedure generated one binary lesion image per participant from which a total lesion volume (in milliliters) map was calculated.

### Hp Genotyping

Blood samples for Hp typing were taken during the physician assessment. Bloods were drawn into an EDTA-containing vacutainer tube and immediately placed in an ice box until handling at Sheba Medical Center. Bloods were centrifuged and serum was stored at −70°C Within up to 6 h from phlebotomy (as recommended), until determination. Hp typing was performed on stored plasma samples by polyacrylamide gel electrophoresis as previously described (24).

**Figure 1:**
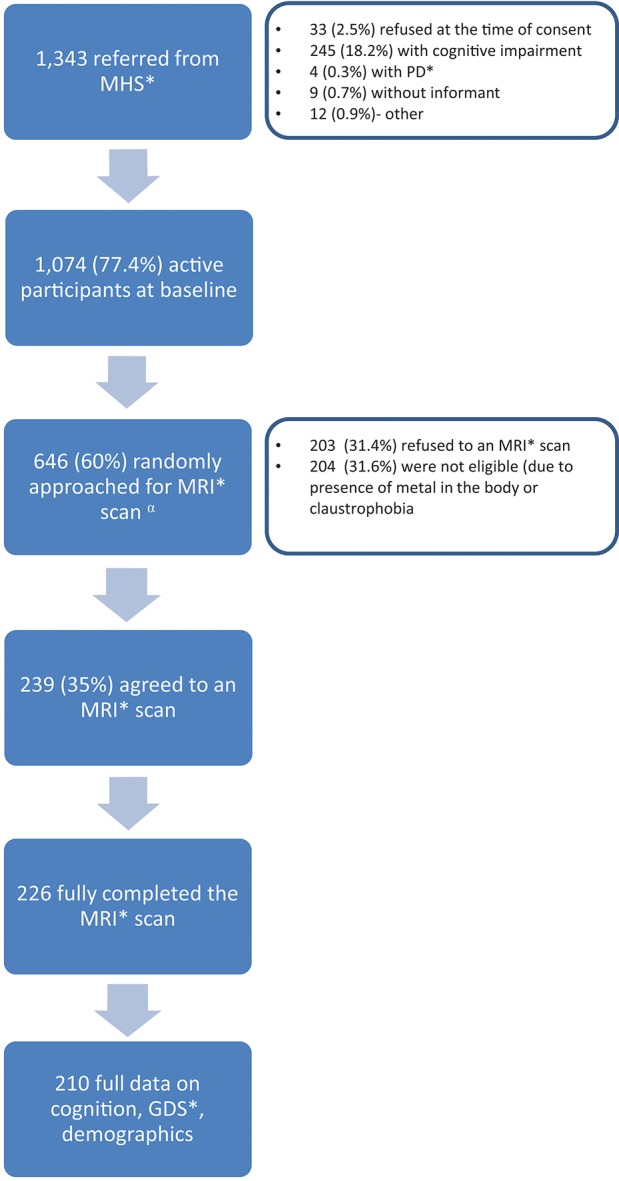
Study flow chart. MHS, Maccabi Health Services; PD, Parkinson's disease; MRI, Magnetic Resonance Imaging; GDS, Geriatric Depression Scale.

IDCD participants included in the present analysis are those with full data on depression, cognition, brain imaging, diabetes, and Hp genotype-related data (Figure 1). This report is based on data collected at IDCD baseline. Subjects' diabetes- related data since 1998 or since entry into the diabetes registry (if after 1998) was available to us through MHS.

### Statistical Analysis

Differences between Hp genotypes on the predictors and brain outcomes were evaluated by Student's *t*-test, and Pearson's chi-square for categorical variables. We compared the genotypes on the relationships of each brain measure with the number of depressive symptoms. Based on similar relationships between Hp 1–1 carriers and the two groups of non-Hp 1–1 (i.e., Hp 1–2 and Hp 2–2) in cognitive outcomes (11), and in order to minimize multiple comparisons, we compared Hp 1–1 carriers to all Hp 1–1 non-carriers. Analysis of the interaction of Hp genotype with number of depressive symptoms in a regression analysis for a brain measure requires three predictors- Hp genotype, the number of depressive symptoms and their product- and tests the product, controlling for its two variables. The regression analysis included additional covariates–age, sex, number of follow up years in the registry (a surrogate of duration of type 2 diabetes), mean level of HbA1c across all measures available in the diabetes registry for each subject, overall cognition (a summary of the *z*-score of all cognitive tests performed in the IDCD study) and total intracranial volume. For Hp 1–1, non-Hp 1–1, and the full sample, we described the relationship of number of depressive symptoms with brain measures using partial correlations controlling for the same covariates. A *p*-value of 0.05 (two sided) was used to determine statistical significance level. For analysis, we used SPSS 22.0 (IBM Corp, Armonk, NY, USA).

## Results

Two hundred and ten subjects with full data on cognition, GDS score, demographic variables and MRI were included in the analysis, 29 with Hp 1–1 (13.8%) and 181 with non-Hp 1–1 genotype. Subjects' mean age was 70.47 (SD = 4.11), 41% were female, with mean duration of follow up in the diabetes registry of 9.46 years (SD = 4.48). Subjects with Hp 1–1 genotype did not differ significantly from non-Hp 1–1 in demographic or cognitive variables, GDS score or in any of the frontal lobe measures, middle temporal gyrus, or white matter hyperintensities volume (see Table 1).

**Table 1:** Sample characteristics^α^.

**Characteristics Mean (SD)**	Hp^*^ 1-1	**Non-Hp 1-1**	***P***	**Total**
*N*	29	181		210
Age mean (*SD*)	70.55 (3.16)	70.52 (4.26)	0.96	70.47 (4.11)
Sex (% female)	41	39	0.82	41
Years of follow up in diabetes registry mean (*SD*)	9.34 (5.04)	9.46 (4.41)	0.90	9.46 (4.48)
Overall cognitive score mean (*SD*)	1.67 (6.66)	2.85 (6.40)	0.36	2.82 (6.44)
GDS^*^ score mean (*SD*)	1.52 (1.78)	1.84 (2.13)	0.45	1.79 (2.06)
Mean HbA1c (mmol/mol)^*^ mean	50.1	49.3	0.66	49.5
Mean HbA1c (%)^*^ mean (*SD*)	6.73 (0.89)	6.66 (0.76)	0.66	6.68 (0.77)
Frontal lobe volume^*^ mean (*SD*)	0.31 (0.03)	0.31 (0.03)	0.36	0.31 (0.03)
Superior frontal gyrus volume^*^ mean (*SD*)	0.30 (0.04)	0.30 (0.03)	0.60	0.30 (0.03)
Middle frontal gyrus volume^*^ mean (*SD*)	0.32 (0.03)	0.32 (0.03)	0.46	0.32 (0.03)
Inferior frontal gyrus volume^*^ mean (*SD*)	0.33 (0.03)	0.33 (0.03)	0.29	0.33 (0.03)
Middle temporal gyrus volume^*^ mean (*SD*)	0.43 (0.04)	0.43 (0.04)	0.48	0.43 (0.04)
White matter hyperintensites volume^*^ mean (*SD*)	11.95 (15.02)	13.25 (15.12)	0.67	12.74 (0.04)
Total intracranial volume^*^ mean (*SD*)	1317.66 (124.91)	1335.78 (138.80)	0.51	1331.62 (136.30)

α *All brain volume measurements are in milliliter units*.

* *Hp, Haptoglobin; GDS, Geriatric Depression Scale; HbA1c, Hemoglobin A1c*.

Overall, for the entire sample, there were no correlations between number of depressive symptoms and regional brain or WMH volume (Table 2). The interaction of Hp genotype with depressive symptoms was significant for overall frontal cortex volume (*p* = 0.01), specifically, the superior frontal gyrus (*p* = 0.01); and approached significance for middle frontal gyrus (*p* = 0.09; see Table 2). There was no interaction for the inferior frontal gyrus (*p* = 0.61) nor with the middle temporal gyrus (*p* = 0.43). To describe the direction of the interactions, we performed partial correlations stratifying for Hp genotype (Hp 1–1 vs. non-Hp 1–1) using the same covariates as in the main analyses. As shown in Table 2, the partial correlation of more depressive symptoms with lower total frontal lobe (*r* = −0.35) and superior frontal gyrus (*r* = −0.42) were substantially stronger for Hp 1–1 carriers than non-carriers. The interaction for white matter hyperintensities volume was also significant (*p* = 0.04) such that depressive symptoms were associated with higher volume of white matter hyperintensities in Hp 1–1 (*r* = 0.45; *p* = 0.03) but not in Hp 1–1 non-carriers (*r* = −0.04; *p* = 0.64).

**Table 2:** Interaction of Hp genotype with depressive symptoms in brain volumes^α^.

**Brain region**		**Partial correlation (p)**	**Partial correlation (p)**	**Partial correlation for interaction (p)**	**Partial correlation for the full sample (p)**
		**Hp 1-1**	**Non Hp 1-1**	**Full sample**	
Frontal lobe	Total	−0.35 (0.11)	0.002 (0.97)	−0.17 (0.01^*^)	−0.07 (0.35)
	Superior gyrus	−0.42 (0.05)	−0.04 (0.58)	−0.18 (0.01^*^)	−0.10 (0.12)
	Middle gyrus	−0.31 (0.15)	−0.03 (0.71)	−0.12 (0.09)	−0.07 (0.29)
	Inferior gyrus	−0.03 (0.91)	−0.04 (0.64)	−0.04 (0.61)	−0.06 (0.40)
Middle temporal gyrus		−0.007 (0.98)	0.01 (0.87)	−0.06 (0.43)	−0.02 (0.80)
White matter hyperintensities		0.45 (0.03^*^)	−0.04 (0.64)	0.15 (0.04^*^)	0.00 (0.99)

α *Controlling for age, sex, intracranial volume, years of follow up in the diabetes registry, mean HbA1c, overall cognitive score*.

* *p < 0.05*.

Since the volume of white matter hyperintensities may be related to smaller frontal lobe volumes (25), we assessed in secondary analyses whether white matter hyperintensities volume mediates the interaction analyses for frontal lobe and superior frontal gyrus, the two frontal lobe measures for which interaction of depression symptoms with Hp genotype was significant, by including it as a covariate. Results were essentially unaffected by the inclusion of white matter hyperintensities (partial correlation for interaction on total frontal lobe *r* = −0.16; *p* = 0.03, and on superior frontal gyrus *r* = −0.16; *p* = 0.02). Similarly, additional secondary analyses added a cardiovascular risk composite to the primary model, which was the first principal component of a factor analysis consisting of systolic and diastolic blood pressure, LDL and HDL, cholesterol, and creatinine (26); or levels of inflammatory markers (CRP and Il-6). Results were mildly attenuated, and remained significant for the interaction of Hp genotype and depressive symptoms on total frontal lobe volume and superior frontal gyrus and trend level for the interaction on white matter hyperintensities volume (Supplementary Table 1).

## Discussion

The present study demonstrates that in elderly patients with T2D, depressive symptoms are modestly associated with larger volume of white matter hyperintensities, and with reduced frontal lobe volume, specifically, superior frontal gyrus, in Hp 1–1 genotype carriers but not in non-carriers. A similar pattern, though not reaching statistical significance, was observed for the middle frontal gyrus. Analysis was adjusted for potential demographic and diabetes-related factors. Adding inflammatory, and cardiovascular potential confounders mildly attenuated the results. These findings may suggest that patients with diabetes who are Hp 1–1 carriers are more susceptible to depression in the context of white matter damage and frontal lobe atrophy, and that the mechanisms underlying depressive symptoms in diabetes may differ by Hp genotype.

Consistent with our results, Maes et al. have shown an association of carrying the Hp 1 allele and hyperhaptoglobinemia with major depression in young to middle aged adults (27). The diabetes status of the sample was not specified, suggesting that the results reflected primarily non-diabetic participants. Our study adds new evidence in several aspects as the study examined the inter-relationships of Hp with number of depressive symptoms (rather than major depression) on brain abnormalities, suggesting potential underlying mechanisms for these relationships. In addition, all our participants were elderly diabetics for whom depressive symptoms are highly prevalent (1, 2), with substantial implications on health, functional capacity, and quality of life (3, 4).

Hp 1–1 genotype has been found to be associated with greater risk for white matter hyperintensities (10), of which the volume is a biomarker for cerebrovascular pathology (28). White matter hyperintensities, in turn, have been demonstrated to be strongly associated with depression, especially in late life (8, 29), supporting the vascular depression hypothesis (8). In the present study, the number of depression symptoms was associated with WMH volume in subjects with Hp 1–1 but not in other Hp genotypes, suggesting that a common mechanism may underlie larger WMH volume and more depressive symptoms in Hp 1–1 carriers. Such a mechanism may be linked to inflammation, which is associated with WMH (30), Hp genotype (31), and depression (32). Since the IDCD study collects CRP and Il-6, we repeated the analysis controlling for these inflammatory factors, and the associations were mildly attenuated, suggesting that inflammation may contribute to the relationship of WMH with depression in T2D subjects carrying the Hp1–1 genotype, but, that additional mechanisms are probably involved. Alternatively, these peripheral markers of inflammation may not fully reflect brain inflammatory processes.

Abnormalities in structure and function of the frontal lobes have been shown to contribute to depression. The dorsolateral prefrontal cortex, formed mainly by the superior and middle frontal gyri, has been shown to be involved in prevention of depression by refuting negative emotions through reappraisal and suppression strategies, and to play a key role in the maintenance of spontaneous optimistic self-evaluative tendencies (16). Accordingly, hypoacitivity of the dorsolateral prefrontal cortex has been demonstrated in depression (16). Elderly subjects with depression had smaller volume of the orbitofrontal cortex compared to non-depressed elderly (33). In the present study, although subjects with Hp 1–1 genotype did not differ in brain volumetrics or in number of depression symptoms, the number of depression symptoms was associated with reduced frontal—specifically superior frontal—gyrus volume. The presence of this relationship only in Hp 1–1 may be attributed to their higher susceptibility to cerebrovascular pathology (34) which in turn, has been associated with brain atrophy, especially in the frontal lobes (25). In the present study, in order to assess whether cerebrovascular pathology mediates the inter-relationships of Hp genotype, depressive symptoms, and frontal lobe volume, we further adjusted the analysis of interaction of Hp genotype with number of depression symptoms on total frontal lobe and superior frontal gyrus volumes for white matter hyperintensities volume, but the results were only mildly attenuated. The vulnerability of subjects with Hp 1–1 genotype to depression symptoms in the presence of frontal lobe atrophy may be attributed to other mechanisms such as compromised endothelial function found in Hp 1–1 carriers (35). Endothelial dysfunction may precede structural vascular changes (36) and lead to brain atrophy (37). It has also been shown to be a risk factor for depression, especially in patients with diabetes (38). This explanation is supported by lower counts of endothelial progenitor cells (involved in endothelial repair) demonstrated in subjects with lacunar strokes carrying the Hp 1–1 genotype (35).

Study strengths include the random selection of participants from the IDCD study, a cohort representative of elderly with type 2 diabetes in Israel; the highly valid diagnosis of type 2 diabetes; the availability of demographic, long-term diabetes-related, genetic and brain imaging variables; and verification of cognitive function, a major confounder for depressive symptoms (39)

The study is limited by its cross-sectional design, precluding identifying the directionality of the relationships found—whether Hp 1–1 carriers are more susceptible for depression in the context of lower frontal lobe volume or higher volume of white matter hyperintensities, or, alternatively, depressive symptoms lead to more brain damage in Hp 1–1 carriers but not in non-carriers. The definition of depression-related outcome in the study is based on the GDS score rather than clinical diagnosis. Although the results are robust and significant, the relatively modest sample size of Hp 1–1 carriers requiring confirmation in future studies. Measurement of endothelial function was not available, preventing evaluation of the contribution of functional cerebrovascular pathology to brain volume and to depression symptoms in different Hp genotypes.

In conclusion: the present study demonstrates interactions between Hp genotype and symptoms of depression on regional brain volumes in elderly non-demented subjects with type 2 diabetes—higher number of depressive symptoms were associated with lower frontal lobe volume and higher volume of WMH in Hp 1–1 carriers but not in non-carriers. The longitudinal phase of the IDCD will elucidate the directionality of the relationships found and will serve as basis for future studies aimed at unraveling the mechanisms involved, in diabetics, and in non-diabetics.

## Ethics Statement

This study was carried out in accordance with the recommendations of the Sheba Medical Center, Maccabi Health Services and the Mount Sinai School of Medicine IRB committees. All subjects gave written informed consent in accordance with the Declaration of Helsinki.

## Author Contributions

ALe and AL: acquisition, analysis and interpretation of data, critical revision of article, and approval of final version. MS, AH, and RR-S: conception and design of study, data analysis and interpretation, critical revision of article, and approval of final version. JS and EM: analysis and interpretation of data, critical revision of article, and approval of final version. RT, GT, and RP: acquisition of data, critical revision of article, and approval of final version. DL: critical revision of article and approval of final version. LS, EG-B, JMS, and BB: interpretation of data, critical revision of article, and approval of final version.

### Conflict of Interest Statement

The authors declare that the research was conducted in the absence of any commercial or financial relationships that could be construed as a potential conflict of interest.
