# Supplementary material for: The Association of Depressive Symptoms With Brain Volume Is Stronger Among Diabetic Elderly Carriers of the Haptoglobin 1-1 Genotype Compared to Non-carriers
Source: Front Endocrinol (Lausanne). 2019 Feb 12;10:68. doi: 10.3389/fendo.2019.00068 (PMC6379325; doi:10.3389/fendo.2019.00068)
Supplement: Supplementary file 1 [file Table_1.pdf]

Supplement table 1 : Interaction of Hp genotype with depressive symptoms in brain volumes[α]

Brain region		Partial correlation (p)	Partial correlation (p)	Partial correlation for interaction (p)	Partial correlation for the full sample (p)
		Hp 1-1	Non Hp 1-1	Full sample	
Frontal lobe	Total	-0.347 (0.146)	0.004 (0.959)	-0.196 (0.020*)	-0.047 (0.513)
	Superior gyrus	-0.425 (0.070)	-0.030 (0.694)	-0.216 (0.014*)	-0.088 (0.216)
	Middle gyrus	-0.311 (0.195)	-0.029 (0.704)	-0.134 (0.115)	-0.058 (0.412)
	Inferior gyrus	-0.006 (0.980)	-0.041 (0.595)	-0.027 (0.740)	-0.040 (0.573)
Middle temporal gyrus		-0.032 (0.896)	0.06 (0.936)	-0.052 (0.537)	-0.011 (0.873)
White matter hyperintensities		0.421 (0.073)	-0.043 (0.581)	0.165 (0.086)	0.003 (0.963)

[α]Controlling for age, sex, intracranial volume, years of follow up in the diabetes registry, mean HbA1c, overall cognitive score, cardiovascular risk composite (consisting of systolic and diastolic blood pressure, LDL and HDL, cholesterol, and creatinine) and levels of inflammatory markers (CRP and Il-6)
